# Online Cognitive Behavioral Therapy for Bulimic Type Disorders, Delivered in the Community by a Nonclinician: Qualitative Study

**DOI:** 10.2196/jmir.2083

**Published:** 2013-03-15

**Authors:** Carrie-Anne McClay, Louise Waters, Ciaran McHale, Ulrike Schmidt, Christopher Williams

**Affiliations:** ^1^Institute of Health and WellbeingMental Health and WellbeingUniversity of GlasgowGlasgowUnited Kingdom; ^2^Institute of PsychiatryKing's College LondonLondonUnited Kingdom

**Keywords:** bulimia nervosa, self-help, cCBT, qualitative research, cognitive behavioral therapy

## Abstract

**Background:**

Cognitive behavioral therapy is recommended in the National Institute for Clinical Excellence guidelines for the treatment of bulimia nervosa. In order to make this treatment option more accessible to patients, interactive online CBT programs have been developed that can be used in the user’s own home, in privacy, and at their convenience. Studies investigating online CBT for bulimic type eating disorders have provided promising results and indicate that, with regular support from a clinician or trained support worker, online CBT can be effective in reducing bulimic symptoms. Two main factors distinguish this study from previous research in this area. First, the current study recruited a wide range of adults with bulimic type symptoms from the community. Second, the participants in the current study had used cCBT with support from a nonclinical support worker rather than a specialist eating disorder clinician.

**Objective:**

To investigate participants’ experiences of using an online self-help cognitive behavioral therapy (CBT) package (Overcoming Bulimia Online) for bulimia nervosa (BN) and eating disorders not otherwise specified (EDNOS).

**Methods:**

Eight participants with a mean age of 33.9 years took part in semi-structured interviews. Interviews were transcribed and analyzed using a 6-step thematic analysis process.

**Results:**

Saturation was achieved, and 7 themes were identified in the dataset. These were: (1) conceptualizing eating disorders, (2) help-seeking behavior, (3) aspects of the intervention, (4) motivation to use the online package, (5) privacy and secrecy with regard to their eating problems, (6) recovery and the future, and (7) participant engagement describing individuals’ thoughts on taking part in the online research study.

**Conclusions:**

Participants suggested that online CBT self-help represented a generally desirable and acceptable treatment option for those with bulimic type eating problems, despite some difficulties with motivation and implementation of some elements of the package.

**Trial Registration:**

International Standard Randomized Controlled Trial Number of the original RCT that this study is based on: ISRCTN41034162; http://www.controlled-trials.com/ISRCTN41034162 (Archived by WebCite at http://www.webcitation.org/6Ey9sBWTV)

## Introduction

Bulimia nervosa (BN) is an eating disorder characterized by recurrent episodes of binge eating and reversing behaviors and has many negative psychological, physical, and social consequences. Cognitive behavioral therapy (CBT) is recommended in the National Institute for Clinical Excellence [1] guidelines for the treatment of bulimia nervosa. In order to make CBT more accessible to individuals who are unable or unwilling to engage in face-to-face therapy, computerized CBT (cCBT) packages have been developed for the treatment of BN. Computerized CBT has been shown to result in improvements in bulimic symptoms when delivered in CD-ROM format [2,3,4] or online [5,6] and can be used in the individual’s own home, in privacy, and at their convenience.

To date, two studies of an online CBT package, “Overcoming Bulimia Online” (OBO) [7] have included a qualitative investigation of users’ attitudes towards and experience of using online CBT. In Pretorius, Rowlands, Ringwood, and Schmidt’s [8] study of adolescents’ experiences of using OBO, the main themes identified included: motivation, support, factors influencing choice of treatment and recovery, and the future. Participants reported choosing online CBT because of its accessibility and flexibility, citing a desire to have autonomy and control over their eating disorder and recovery. Many participants indicated that they considered cCBT as a first step in their treatment and perhaps as a first step towards additional treatment options due to increased confidence in discussing their eating problems, but they reported that low motivation was a barrier to using this intervention.

Sanchez-Ortiz et al [9] more recently carried out a qualitative study of students’ experiences of OBO. Interviewees cited flexibility, confidentiality, and ease of use as positive aspects of the online package. Interestingly, many participants discussed other treatments they had accessed and compared these with their experience of OBO. Participants indicated that the online CBT package was more structured than counseling. Also, some participants expressed feelings of apprehension with regard to approaching their GP about their eating problems. This may be one of the reasons that some individuals see cCBT as a more desirable treatment option. However, that study and the randomized controlled trial (RCT) from which participants were drawn focused on students [6]. Students are a highly selected group educationally, in terms of age, their access via universities to high-speed Internet, and their information technology literacy.

The current study aimed to add to this growing body of literature regarding a wider population of user’s experiences of using a cCBT package for bulimia nervosa. Two main factors distinguish this study from previous research in this area. First, the current study recruited a wide range of adults with bulimic type symptoms from the community. This meant that a more representative sample of the population could be recruited. The two studies previously mentioned recruited individuals who were highly likely to favor this online treatment approach: students and adolescents. Second, in contrast to the studies of Pretorius et al [8] and Sanchez-Ortiz et al [9], all participants in the current study had used OBO with support from a nonclinical support worker rather than specialist eating disorder clinicians. This is significant since, having similarly favorable results, such as when the support is provided by specialists, would support the view that low intensity CBT can be delivered and supported by trained but nonspecialist support staff. This would save resources in services and aid the increase in access to psychological therapies, a main treatment target in the National Health Service.

Therefore, the purpose of this qualitative study was to investigate whether similar themes emerged with this new population. The study also aimed to investigate reasons for choosing the self-help approach and to compare attitudes towards cCBT with other treatments that participants had accessed in the past. A final aim was to determine participants’ attitudes towards taking part in a predominantly online research study where recruitment, questionnaires, and follow-up assessment were carried out using online web pages and emailed links to online evaluations.

## Methods

### Recruitment

All participants invited for interview were taking part in an RCT of Overcoming Bulimia Online. These participants had been recruited to take part in an initial survey about attitudes towards online self-help for eating disorders [10] and were then screened and invited to take part in the RCT. Various community-based recruitment methods were employed in the study. Advertisements were placed on mental health websites, in mental health newsletters, and an article about the study appeared in an eating disorders magazine (*Anorexia and Bulimia Care*). Posters were also placed in public places such as libraries, gyms, universities, colleges, and student counseling services. Additionally, flyers giving details of the study were put into university students’ orientation packages.

### Participants

Ten months into the RCT of OBO, 55 participants who had had access to the online package for at least 10 weeks were grouped according to how many sessions of the package they had completed—defined as low users (0-2 sessions), medium users (3-5 sessions), and high users (6-8 sessions) (the OBO package contains 8 sessions in total). A purposive sampling approach was taken with 12 participants from each group who were randomly selected to be invited for interviews using the randomization function in SPSS 12.

Nine participants agreed to take part in the interviews. One interview could not be transcribed due to a poor recording, so 8 interviews were used in the final analysis. As expected, the number of sessions completed had increased by the time of interview as participants still had access to the online package, therefore the sample contained no low users (0-2 sessions), 2 medium users (3-5 sessions), and 6 high users (6-8 sessions). The mean number of sessions completed was 6.4 out of the 8 session online course. All participants were women; the mean age of the sample was 33.9 years (range = 28-50 yrs). All individuals met the criteria for Bulimia Nervosa or Eating Disorder Not Otherwise Specified (EDNOS), as established prior to entry into the RCT using the Eating Disorders Examination [11]. Duration of their eating disorders ranged from 2 years to 30 years; the mean was 16.6 (SD 8.6).

### Procedure

#### The Online Intervention: “Overcoming Bulimia Online”

**Figure 1:**
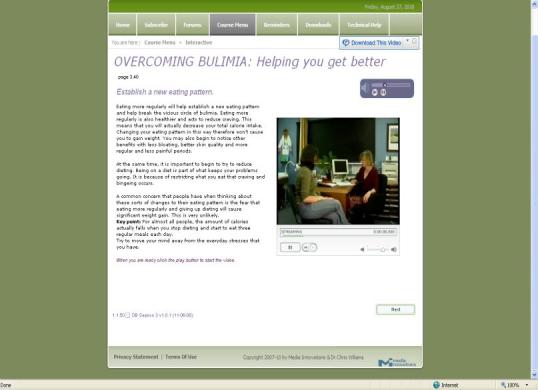
Screenshot from OBO, Session 3: "How do I change?"

Participants interviewed had been given access to the online CBT based self-help package OBO as part of an RCT. The package contained 8 interactive sessions that aimed to change users’ thoughts, feelings, and behavior with regard to food. As illustrated in Figure 1, the sessions contained written text and video clips, which were supplemented by audio information and instructions. The 8 topics covered in the package were: (1) Introduction and What is Bulimia?, (2) Understanding why I have Bulimia, (3) How do I change?, (4) The role of thoughts in Bulimia, (5) Assertiveness and increasing activity, (6) Problem solving, (7) Living Life to the Full, and (8) Planning for the future and review of what I have learned.

Participants were encouraged to work through the sessions independently in their own time and chose to receive weekly telephone, email, or text message support. These 8 support sessions were provided along with the online package by a trained self-help support worker. The support worker was a research assistant with experience of working in the area of eating disorders and guided CBT research. She had written guidelines on how to deliver guided self-help and was supervised by the clinical lead of the project (CW). At the beginning of the intervention, 3 of the participants chose to receive their support sessions over the telephone, 4 requested email support, and 2 opted for text message support to their mobile phones. The support time varied depending on the extent to which the participant engaged with the support, ie, replied to the support emails.

**Figure 2:**
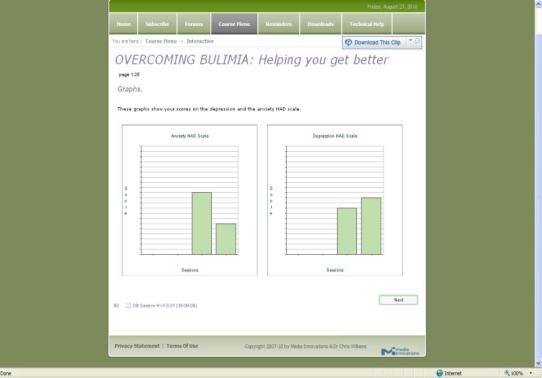
Screenshot of the mood rating graphs in Overcoming Bulimia Online.

At the end of each module, participants were asked to complete various homework assignments such as using the anxiety control training audio daily until the next session, implementing some of the rules of healthy eating, and keeping food, thought, and activity diaries. Additionally, participants were asked to rate their mood and give information about their bingeing and vomiting at the beginning of each session. This information was recorded and presented in a graph that could be viewed by the participant and any authorized support workers, researchers, or clinicians (see Figure 2).

#### The Interviews

Following informed consent, semi-structured interviews were carried out by C-A M over the telephone and were recorded using a digital recording device. A question guide was devised by the research team to probe issues such as participants’ reasons for choosing the self-help approach, their experience of using the online package, motivational issues while using the package, how the intervention compared to other treatments they had accessed in the past, and aspects of the online research process. The mean duration of the interview was 19.4 minutes.

### Analysis

Interviews were recorded, transcribed verbatim, and analyzed using the 6-step inductive thematic analysis approach outlined by Braun and Clark [12]. This method of analysis was chosen because the identified themes are data driven. As such, themes are identified without trying to fit them into a pre-designed coding framework.

Two researchers (C-A M and CM) carried out the coding and identification of themes limiting the subjective nature of the analysis, thus increasing the validity. Both researchers independently generated initial codes for all interesting extracts. Using these codes, themes were then searched for. Once this stage was complete, the analyzers discussed the themes that they had identified and agreed upon the final themes that would be used. All coded extracts were then put into the appropriate themes and subthemes. In some cases, extracts were themed differently by the researchers; C-A M discussed these with a third member of the research team (LE) who was experienced in the process of qualitative analysis and then made the final decision on where these extracts belonged. All themes were reviewed and the extent to which they encapsulated the data was judged.

## Results

Analysis of transcripts identified seven main themes, as illustrated in Table 1.

**Table 1:** Themes and subthemes identified in the semi-structured interviews.

Themes	Subthemes
Conceptualizing eating disorders	Impact of and feelings about bulimia
	Perceptions of eating disorders/people with eating disorders
	Acknowledgement of/acceptance of the problem
Help-seeking	Past experiences
	Barriers to help
	Reasons for choosing self-help
	Prior knowledge
The intervention	Support worker
	Positive aspects
	Negative aspects/difficulties
Motivation	Aids
	Challenges
Participant engagement	Opportunity to help self and others
	Experience as an online research participant
Progress and recovery	Improvements in bulimic symptoms
Privacy	Secrecy
	Talking about bulimia

### Conceptualizing Eating Disorders

Participants stated that their eating problems had resulted in many negative consequences including low self-esteem, poor self-concept, a negative view of the future, and concerns about the impact of the disorder on their family. Participants also spoke of feelings of isolation and shame associated with BN, and there was a sense that their eating problems were a significant burden in their lives: “I thought I was just a horrible, horrible person and all I deserved was to be who I am”. [P9]

Several statements were made relating to perceptions of people with eating disorders. There was a sense that views such as these were damaging and affected confidence in help-seeking and talking about eating problems: “...there’s a standard joke that people who are bulimic are failed anorexics...and that’s an extra thing that you carry around with you”. [P1]

Although participants had very negative feelings about their eating problems, some interviewees expressed relief in having someone acknowledge their eating problems: “I could take a deep breath and think oh my god (laughs) this is real. Because of all those years of people saying to me oh you’re just jumping on the band wagon and all the rest of it”. [P1]

The statements by these participants seemed to indicate a turning point and a sense that now their problems had been acknowledged by another person (the researchers), that they then could accept them and start to tackle these problems.

### Help-seeking

All participants discussed their past help-seeking and their experience of various treatment and support options including: their GP, formal therapy, self-help books, and support groups. Negative experiences described included: difficulty in understanding self-help books, unsatisfactory support services, and the feeling that their problems were dismissed or inadequately dealt with: “...that I’ve tried over the years is talking to my GP, various GPs about it and just been fobbed off so... Yeah I’ve been turned, turned away by I think it’s 4 GPs who’ve all said it’s not a problem”. [P1]

The most common barrier identified by the majority of participants was the lack of access to treatment through the NHS. There was a general sense of low expectations of the NHS and a portrayal that these individuals felt that they had to arrange help for themselves: “And also because there’s not much help out there for like, you know if you go to your doctors there’s a long waiting list, that’s if you get help, so it’s just wanting to recover and get whatever help is available...”. [P2]

Practical issues and readiness for change were also identified as barriers to accessing help in the past. Travelling to an eating disorders center or therapy session, taking time off work, and thus having to disclose the eating disorder was identified as a problem. Individuals voiced being reluctant to seek help or had chosen to manage their problems alone, often due to the common feelings of shame and embarrassment known to be associated with the disorder: “I’ve tried, er, self-help books, you know looked at books in the library...Em, I never really wanted to take them out because of the embarrassment of asking for them over the counter”. [P1]

The issue of prior knowledge about eating disorders and their treatments was raised by a few interviewees. The extent to which participants understood eating disorders varied with one participant expressing confusion concerning the causes of her BN and another stating that despite having a sound knowledge of the problem, she was unable to use this information to help herself: “I used to work with adolescents and the adolescents either had eating disorders or were self-harmers so I had a lot of knowledge that I never applied to myself”. [P1]

Participants outlined various reasons for choosing self-help. There was a sense that the self-help approach seemed like a desirable alternative to traditional face-to-face treatment, primarily because of the private, convenient, flexible, and anonymous nature of the treatment. Autonomy and the ability to deal with these problems independently were also highlighted as attractive aspects of self-help materials: “you didn’t have to sit there with a patronising (pause) um person, being judged every week on whether you follow what she actually said to you or not”. [P7]

Two participants also stated that feelings of desperation led them to seek help on the Internet, an indication again that perhaps other routes of accessing support for their eating problems had been unhelpful or undesirable. The following statement highlights the serious situation that these undiagnosed and untreated individuals are in and the importance of community initiatives in order to identify and support such individuals: “I was really, really desperate...when people are looking through Internet websites for online help...it’s the question between living and dying really and that’s where I was”. [P9]

### The Intervention

Interviewees were largely positive about the online package. Many participants stated that OBO was helpful because it increased their understanding of their problems. Participants also said that the intervention was very specific and gave them useful skills and tools that they could use to improve their situation. There was a general indication that the package was educational and easy to understand: “It helps you tackle other stuff as well, and being assertive, it sort of just really—it teaches you the whole lot of it, if you get what I mean. It’s just not one thing, it’s lots of little things I think now that cause it”. [P8]

Participants expressed that a particularly useful element of the package was the “Letter from the future” and also the “challenging unhelpful thoughts” session with the thought flashcards: “I think the most helpful idea was the idea of the letters from the future...actually just thinking about it, in 10 years time do I actually still want to be in the same position and thinking about the things that I might be missing out on”. [P6]

Again, participants highlighted the notion of privacy and autonomy as being a positive aspect of such a treatment. The fact that the treatment could be accessed without others knowing about it was cited as a main advantage of OBO. The convenient and quickly accessible nature of the package was also outlined as an advantage: “...it was good because it was convenient so uh huh you could fit it into, you know, the rest of your life”. [P5]

Participants also mentioned a number of difficulties or challenges that they faced when using the self-help package including technical difficulties, a lack of support, and motivation. A key difficulty identified by participants was the fact that the package was not tailored enough to their needs. Participants described the fact that some content was not new to them, the package was designed to meet the needs of a group of people with BN rather than the individual, and one individual got the impression that the package was aimed at younger users: “...if you click back by accident you end up losing everything so that’s a bit rubbish” [P7] and “there was probably was a need for a personal element but em, a bit more tailored like em, like that fitted somebody who was 20 years down the line”. [P4]

However, it was acknowledged that this difficulty was perhaps an inevitable aspect of using an online self-help package. Participants also expressed difficulties in implementing aspects of the package such as a healthy eating regime, using the anxiety control training, and challenging unhelpful thoughts: “I did start to kind of engage with a healthier eating pattern but I immediately started to put on weight so that was, that really put me off”. [P6]

Other difficulties include the lack of discretion of the web pages of OBO with participants saying they could not use the package unless they were alone because the topic of the package was very obvious: “...the package itself when you go online it, it was quite clearly that it is to do with bulimia, bulimia so I couldn’t use it a lot of the time”. [P4]

This links closely to another difficulty experienced by some participants: lack of privacy to use the package. Two participants said that they found it difficult to get time alone to use the online package without people such as family members interrupting them.

Interviewees referred to the workload involved in completing the package and the pace at which they completed the sessions. Many said that they were unable to complete the sessions at the pace suggested, one session per week ,and although the majority of participants referred to the workbooks, they were often not actively used: “I think it’s quite a bit to be involved in the package...found er um, almost impossible to do one a week” [P6] and “the thought of having to fill more books in and logs and things when you do that sort of thing all day it just, it’s just a complete turn off”. [P4]

Participants stated that the support worker was a valued element of the intervention and that it was good to have someone there to help with their progress through the package without judgment: “...she has made a huge huge difference so I appreciate all the chats I’ve had with her and she’s good...yeah I think it’s made, it made a massive massive difference”. [P7]

Additionally, email support was considered an effective means of receiving the support. Participants also referred to the flexibility of the support. One stated that it was helpful to change their support medium when required. Others said that they wish they had taken advantage of this flexibility, perhaps indicating that this option should have been promoted more: “I think that it might have been better for me for sometimes to actually email and say I want to talk over the phone about this”. [P5]

However, some participants outlined the fact that the weekly contact from the support worker made them feel pressured to complete the sessions, and one participant indicated that she did not engage in the support sessions because she felt guilty when she had not made any progress with the package: “I didn’t find it supportive because it asked about 5 or 6, if not more questions of which I couldn’t answer any of them because I’d only just literally been given access to the online package”. [P4]

Another participant said that she did not feel that the support helped her progress, although she did appreciate the texts from the support worker: “Em, that, that didn’t really help me either way...I think (the support worker) just used to text me everyone now and then to see if I was ok. Which was nice”. [P8]

Some participants suggested that it would be helpful to have continued support after the 8-10 week intervention period. They said that it would beneficial to have follow-up sessions with their support worker in the months following the intervention to discuss how things are going and perhaps keep them on track with the improvements they had made: “You know like not completely cut off the contact but...Yeah. But stay in touch even for a longer period of time”. [P9]

### Motivation

A general feeling of low motivation was portrayed by some participants, indicating that this was a major obstacle to their progression with the intervention: “Well, er actually, it was easier to just not really do it”. [P7]

Two individuals said they had to work on getting the motivation to start the package: “...it’s been quite hard to sort of em, I suppose, motivate myself... because I always thought that once I got treatment, it would, you know, getting the treatment would be the difficult thing and then once I started, you know, it would be easy”. [P5]

A lack of support was also cited as something that affected participants’ motivation in using the package. Some individuals stated that they would have benefited from more human interaction.

Interviewees cited a wide variety of things that aided them in maintaining motivation while using OBO. The support worker was the most commonly cited aid. Participants said that the support worker helped to reassure them about various issues relating to the package and helped them move past difficulties and continue through the package: “I think if it hadn’t been for (the support worker) at that point. I would have thought that it’s not it er is obviously not working, it’s not worth persevering with”. [P1]

Knowing that there was someone there who knew that they were using the package and asking how things were going helped some individuals to maintain their motivation: “you’ve still got that person in the background who’s wanting you know, wanting to know what’s happening so that’s motivation”. [P1]

Other aids highlighted by interviewees included: telling family members about their use of the package, the study newsletter, completing the follow-up questionnaires, thinking about the “Letter from the future” and actually completing the sessions. Planning and goal setting was identified as important by some individuals, with some participants expressing difficulties in completing the sessions, acknowledging that planning their next session was really needed to make any progress: “...you’ve got to just try and be determined haven’t you, set yourself the goals to do it. (hesitation) mmm you know work towards achieving those goals”. [P2]

Finally, some participants were motivated to use the package because they saw it as an opportunity for change and to address their eating problems: “I just thought, if I don’t take this opportunity and sort of, em, give it my best shot, mm huh then I was going to go back to how I was”. [P5]

### Participant Engagement

This theme relates to participant’s experiences of taking part in the online research study. Many participants saw taking part in the study as a significant opportunity to gain access to treatment with many participants portraying a significant desire for change in their lives: “I thought er this is great, an opportunity you know to take part in the study, to do something that I er I wouldn’t normally be able to access”. [P1]

Participants also expressed the fact that they wanted to make a contribution to the research and aid the development of treatment for the future, to help others with similar problems: “...you’re kind of involved in a research study which means that you’re kind of doing something which might benefit other people as well”. [P5]

The online nature of the research process was generally acceptable to participants with many participants saying that the website was easy to use and that completing questionnaires online was convenient and economical: “I think in this day and age online is a good thing”. [P7]

### Privacy and Secrecy

The concepts of privacy and secrecy were pertinent throughout the dataset and related to many of the themes. As outlined, the desire for privacy with regard to their eating problems affected help-seeking behavior and the preference for the self-help approach. The private nature of OBO was cited as a significant advantage of this intervention. However, the desire for their eating problems to be kept hidden meant that some individuals had limited external support when their support sessions ceased with some saying that they had no one else to talk to about their problems. This had an impact on their motivation to address their problems. Additionally, as previously described, maintaining secrecy with regard to their eating problems had an impact on participants’ use of the online sessions and accompanying materials because they did not want others to see them: “I don’t actually have anybody else, there’s nobody else in my life day to day that I’ve told about this”. [P6]

This desire for privacy and secrecy was acknowledged as damaging by one participant. This participant said that having used the intervention, she is now more open to talking about her eating problems: “And there’s something about you know it being hidden and awful that it’s not helpful isn’t it...It just feeds itself”. [P1]

### Progress and Recovery

Many participants said that they had made noticed improvements in their bulimic symptoms and behavior with regard to food and eating: “I’m nearly recovered now em...I think it was sometime in June that I last made myself sick and even that was a one off”. [P8]

Others had identified more subtle differences in their behavior by applying some of the skills they had learned from the package. For example, one participant found the Rules of Healthy Eating particularly helpful: “...actually saying, ‘that’s your meal over’ kind of thing yeah and I’ve done that quite a few times without really thinking of it”. [P9]

However, there was some indication that the package perhaps could not completely solve eating problems with some suggesting that more time and support are required to address these problems. Related to this point, some participants mentioned other helpful forms of support that they had accessed for their mental health problems including high intensity specialist psychotherapeutic help such as a therapist, Cognitive Analytic Therapy, and a social worker. “..it’s not just something that you can get over in 8 weeks”. [P7]

Finally, one participant said that the package helped her to gain a more internal locus of control with regard to her eating disorder and her recovery and made her take a more autonomous approach to addressing her problems: “I realised throughout my recovery that there is no one else that can help me but myself that was just, just an eye opening moment...” [P9]

## Discussion

### Summary and Discussion of Key Findings

This qualitative study is a significant contribution to the growing body of literature regarding participants’ experiences of using the online self-help package “Overcoming Bulimia Online” for bulimia nervosa. These participants were recruited from the wider community rather than a specialist eating disorders setting or student setting, and all participants were supported by a nonspecialist for the first time in a BN research study of this kind. This method has previously been successful in a study of cCBT for depression [13]. Participants expressed a range of attitudes towards help-seeking, and in particular, their negative past experiences support statements made in previous studies. As in Sanchez-Ortiz et al’s [9] study, participants indicated that many previous sources of support had been unsuccessful including: counseling, self-help books, CBT, and GP visits. There was a general sense of negativity regarding accessing traditional treatments, an awareness of long waiting lists, and one participant reported being turned away or “fobbed off” by a GP. This experience supports the statistics from a *beat* (UK eating disorders voluntary sector organisation) survey [14] in which only 15% of respondents felt that their eating problems were understood by their GP. Thus taken together the findings highlight the fact that perhaps there needs to be more information and training available to primary care workers.

This lack of faith in traditional treatments and participants’ negative past experiences influenced the decision to try the online guided CBT self-help approach. Additionally, the private, accessible, and convenient nature of online CBT was attractive to interviewees. These reasons for choosing self-help have been cited in previous studies of computerized CBT [8,15,16] and seem to represent a key attraction of the self-help approach for those who do not wish to pursue traditional routes of treatment.

Similar to the findings of previous studies of the OBO package [8,9], the support that participants received from their support worker while using OBO was commented on frequently and in the present study was seen as a core element of the intervention for the majority of participants, particularly in relation to maintaining motivation. This is important as the support was not provided by a clinician, as in previous studies, but was still considered as highly helpful and desirable by the majority of participants. This highlights the fact that support can be effectively delivered by trained nonspecialist health care workers, overcoming the problem of the lack of CBT therapists in many areas. This supports the view that low intensity CBT can be delivered by individuals who have had self-help training rather than specialist CBT training, therefore increasing access to psychological therapies and reducing the cost of psychological interventions for bulimia nervosa.

Another significant finding was that some participants expressed the desire for follow-up support sessions following the block of 8 support sessions they received while working through the package. There was a sense that some participants felt abandoned when the support ceased, and follow-up sessions may be needed to help keep participants on track with their progress, particularly for those with little or no social support. This suggestion for how to improve the support provided is valuable and could be easily implemented in future delivery of the intervention.

Motivation was widely cited as a problem in using the online package with many individuals saying that it was often difficult to maintain drive and determination to engage with the package. The support worker, goal setting, and seeing themselves making progress were all cited as increasing motivation. However, factors such as a lack of progress with the sessions, the feeling that the intervention was not working, and the fear of gaining weight all hindered motivation to engage with the intervention. The latter factor is significant as it is something that can be tackled within self-help packages. It is possible that an additional session early on in the package addressing body image and weight concern may specifically help to alleviate such concerns and may prevent such worries from interfering with the individual’s engagement with the later sessions and their implementation. The site was overall considered to be user-friendly, despite some reported minor technical difficulties with navigation.

The findings showed that the desire for privacy and secrecy had a detrimental impact on participants’ help-seeking, their use of the intervention, and the support they could seek from family and friends. This finding highlights the often cited problems faced by individuals with eating disorders such as shame, embarrassment, and the attached stigma. This may be a reason that individuals with eating problems often do not seek the help they need. This shame and embarrassment may also mean that individuals are less willing to seek support from significant others such as carers or health workers when they do take the step to seek help and engage with an intervention. This supports the need for community-based initiatives in order to identify and engage such individuals who do not wish to access help through traditional channels. Future research should examine these feelings of shame and investigate how they can be overcome to allow individuals to seek the help they need, whether it is through traditional (NHS) or nontraditional routes including direct self-referral or interventions offered via the voluntary sector such as *beat*.

Overall participants expressed positive attitudes towards taking part in the study and saw it as a valuable opportunity to not only help themselves but also to contribute to the research and possibly help others with similar problems. As in the two previous qualitative studies of OBO [8,9], some participants outlined the fact that they had noticed improvements in their eating problems. This supports the use of online CBT as a first step in the treatment of even chronic bulimic type eating problems, as recommended in the NHS National Institute for Health and Clinical Excellence guidelines [1].

The online nature of the research project was popular with many participants saying that they felt it was easy to participate and that completing the questionnaires was convenient and efficient. This suggests that this online format could be an efficient, effective, and acceptable method of recruitment, screening, and data collection, especially in a group of individuals who perhaps want the entire research process and intervention to remain remote and anonymous.

### Limitations

The study had some limitations. First, the sample size was relatively small, and the randomization for the interviews took place before the end of recruitment meaning those who entered later could not be interviewed. However, as it is a qualitative study in which a small sample size is expected, the important issue is one of saturation rather than generalizability, and saturation was achieved. Also, the sample consisted of purely community-based users who were actively seeking help and chose to take part in a study on cCBT and therefore may have more positive attitudes towards the approach than those seeking specialist therapies. This may affect the relevance of the findings in a clinical setting. The aim of the study was, however, to investigate experiences of community-based users and their engagement with an online self-help package with remote support. This delivery model may serve as a valuable support option for individuals in community and voluntary sector setting. Future research should assess uptake and efficacy of cCBT in clinical practice in order to gain a more complete knowledge of the potential of cCBT as a treatment for BN.

Despite these limitations, this study adds to the growing body of research in this area, first, because the participants had been recruited directly from the community through various avenues. The sample had a wider age range than previous qualitative studies, and the mean age was also higher in this study than previous studies, providing the opinions of an older group of user with the majority having chronic eating problems. Additionally, community-based recruitment meant that a variety of individuals entered the study, which means that the results may be more relevant to the general population than in previous studies of young people and students. The study provided further qualitative data from individuals who may not normally access treatment due to the lack of services available to them or the desire for privacy in relation to their eating disorders.

### Conclusions and Implications

This study provides valuable information regarding participants’ views of taking part in an online research study and may influence the implementation of future community based projects, within the area of mental health. The information regarding the factors that aid and hinder participants’ motivation in using online self-help could also be used in the development of future online self-help strategies in order to maximize adherence and health and mental health-related outcomes. Finally, the study was the first of its kind to include individuals who had been involved in a guided self-help intervention for BN that used solely nonspecialist support workers and has established the acceptability of this type of support.

In the NHS, this intervention could be tested and, if successful, applied by staff members including assistant psychologists, nursing staff, or other members of staff trained in the delivery of self-help materials. This approach would save resources, reduce waiting lists, and therefore increase access to psychological therapies to those who are suitable for and willing to use cCBT resources. The guided self-help model therefore has the potential to improve productivity and facilitate early intervention. The implementation of cCBT will also aid the achievement of the NHS targets as it allows the treatment of high numbers of patients with minimal therapeutic input. However, the results of a recent survey of Scottish NHS boards indicated that NHS policy and infrastructure may not be fully in place to support to implementation of cCBT in many areas [17]. Overall, most of the health boards possess the required software to use cCBT programs. However, the majority of NHS health boards reported that they lack dedicated computers for patient use, hence access to cCBT at NHS sites is limited. Additionally, local policy in the majority of boards prevent staff from routinely contacting patients via email, Skype, or instant messenger, making the delivery of short efficient support sessions difficult.

For cCBT to be successfully delivered in the treatment of BN within a guided support model, as recommended by national guidelines, dedicated patient computers should be provided to allow access to online interventions. Additionally, IT policy should allow staff to support patients in convenient ways such as via email or live chat, and procedures need to be developed to allow this within confidential and appropriate clinical support. These measures would increase access to reputable, evidence-based, and expertly supported online packages such as OBO; this would serve as an alternative to poor quality mental health websites and potentially harmful “pro-ED” websites that many individuals, in the absence of specialist treatment, with eating problems encounter online [18].

## Abbreviations

BN: bulimia nervosa
CBT: cognitive behavioral therapy
cCBT: computerized cognitive behavioral therapy
ED: eating disorder
EDNOS: eating disorder not otherwise specified
OBO: Overcoming Bulimia Online

## References

[1] National Collaborating Centre for Mental Health (2004). Eating Disorders: Core Interventions in the Treatment and Management of Anorexia Nervosa, Bulimia Nervosa, and Related Eating Disorders.

[2] Bara-Carril N, Williams CJ, Pombo-Carril MG, Reid Y, Murray K, Aubin S, Harkin PJ, Treasure J, Schmidt U (2004). A preliminary investigation into the feasibility and efficacy of a CD-ROM-based cognitive-behavioral self-help intervention for bulimia nervosa. Int J Eat Disord.

[3] Murray K, Schmidt U, Pombo-Carril M, Grover M, Alenya J, Treasure J, Williams C (2007). Does therapist guidance improve uptake, adherence and outcome from a CD-ROM based cognitive-behavioral intervention for the treatment of bulimia nervosa?. Computers in Human Behavior.

[4] Schmidt U, Andiappan M, Grover M, Robinson S, Perkins S, Dugmore O, Treasure J, Landau S, Eisler I, Williams C (2008). Randomised controlled trial of CD-ROM-based cognitive-behavioral self-care for bulimia nervosa. Br J Psychiatry.

[5] Pretorius N, Arcelus J, Beecham J, Dawson H, Doherty F, Eisler I, Gallagher C, Gowers S, Isaacs G, Johnson-Sabine E, Jones A, Newell C, Morris J, Richards L, Ringwood S, Rowlands L, Simic M, Treasure J, Waller G, Williams C, Yi I, Yoshioka M, Schmidt U (2009). Cognitive-behavioral therapy for adolescents with bulimic symptomatology: the acceptability and effectiveness of internet-based delivery. Behav Res Ther.

[6] Sánchez-Ortiz VC, Munro C, Stahl D, House J, Startup H, Treasure J, Williams C, Schmidt U (2011). A randomized controlled trial of internet-based cognitive-behavioral therapy for bulimia nervosa or related disorders in a student population. Psychol Med.

[7] Williams CJ, Aubin SD, Cottrell D, Harkin PJR (1998). Overcoming bulimia: A self-help package (computer software).

[8] Pretorius N, Rowlands L, Ringwood S, Schmidt U (2010). Young people's perceptions of and reasons for accessing a web-based cognitive behavioral intervention for bulimia nervosa. Eur Eat Disord Rev.

[9] Sánchez-Ortiz VC, House J, Munro C, Treasure J, Startup H, Williams C, Schmidt U (2011). "A computer isn't gonna judge you": a qualitative study of users' views of an internet-based cognitive behavioral guided self-care treatment package for bulimia nervosa and related disorders. Eat Weight Disord.

[10] McClay C-A, Ewan L, Schmidt U, Williams C (2011). Attitudes towards self-help for eating disorders. Proceedings of the 39th British Association for Behavioral and Cognitive Psychotherapies' Annual Conference.

[11] Fairburn CG, Cooper Z, O'Connor M, Fairburn CG (2008). Eating Disorders Examination. Cognitive Behavior Therapy and Eating Disorders.

[12] Braun V, Clarke V (2006). Using thematic analysis in psychology. Qualitative Research in Psychology.

[13] Titov N, Andrews G, Davies M, McIntyre K, Robinson E, Solley K (2010). Internet treatment for depression: a randomized controlled trial comparing clinician vs. technician assistance. PLoS One.

[14] beat beat.

[15] Carrard I, Rouget P, Fernández-Aranda F, Volkart AC, Damoiseau M, Lam T (2006). Evaluation and deployment of evidence based patient self-management support program for Bulimia Nervosa. Int J Med Inform.

[16] Murray K, Pombo-Carril MG, Bara-Carril N, Grover M, Reid Y, Langham C, Birchall H, Williams C, Treasure J, Schmidt U (2003). Factors determining uptake of a CD-ROM-based CBT self-help treatment for bulimia: patient characteristics and subjective appraisals of self-help treatment. Eur Eat Disorders Rev.

[17] Kenicer D, McClay CA, Williams C (2012). A national survey of health service infrastructure and policy impacts on access to computerised CBT in Scotland. BMC Med Inform Decis Mak.

[18] Peebles R, Wilson JL, Litt IF, Hardy KK, Lock JD, Mann JR, Borzekowski DL (2012). Disordered eating in a digital age: eating behaviors, health, and quality of life in users of websites with pro-eating disorder content. J Med Internet Res.

